# A Deep Learning Method to Automatically Identify Reports of Scientifically Rigorous Clinical Research from the Biomedical Literature: Comparative Analytic Study

**DOI:** 10.2196/10281

**Published:** 2018-06-25

**Authors:** Guilherme Del Fiol, Matthew Michelson, Alfonso Iorio, Chris Cotoi, R Brian Haynes

**Affiliations:** ^1^ University of Utah Department of Biomedical Informatics Salt Lake City, UT United States; ^2^ Evid Science Los Angeles, CA United States; ^3^ InferLink Corporation Los Angeles, CA United States; ^4^ Department of Health Research Methods, Evidence, and Impact McMaster University Hamilton, ON Canada; ^5^ Department of Medicine Faculty of Health Sciences McMaster University Hamilton, ON Canada; ^6^ Health Information Research Unit McMaster University Hamilton, ON Canada

**Keywords:** information retrieval, evidence-based medicine, deep learning, machine learning, literature databases

## Abstract

**Background:**

A major barrier to the practice of evidence-based medicine is efficiently finding scientifically sound studies on a given clinical topic.

**Objective:**

To investigate a deep learning approach to retrieve scientifically sound treatment studies from the biomedical literature.

**Methods:**

We trained a Convolutional Neural Network using a noisy dataset of 403,216 PubMed citations with title and abstract as features. The deep learning model was compared with state-of-the-art search filters, such as PubMed’s Clinical Query Broad treatment filter, McMaster’s textword search strategy (no Medical Subject Heading, MeSH, terms), and Clinical Query Balanced treatment filter. A previously annotated dataset (Clinical Hedges) was used as the gold standard.

**Results:**

The deep learning model obtained significantly lower recall than the Clinical Queries Broad treatment filter (96.9% vs 98.4%; *P*<.001); and equivalent recall to McMaster’s textword search (96.9% vs 97.1%; *P*=.57) and Clinical Queries Balanced filter (96.9% vs 97.0%; *P*=.63). Deep learning obtained significantly higher precision than the Clinical Queries Broad filter (34.6% vs 22.4%; *P*<.001) and McMaster’s textword search (34.6% vs 11.8%; *P*<.001), but was significantly lower than the Clinical Queries Balanced filter (34.6% vs 40.9%; *P*<.001).

**Conclusions:**

Deep learning performed well compared to state-of-the-art search filters, especially when citations were not indexed. Unlike previous machine learning approaches, the proposed deep learning model does not require feature engineering, or time-sensitive or proprietary features, such as MeSH terms and bibliometrics. Deep learning is a promising approach to identifying reports of scientifically rigorous clinical research. Further work is needed to optimize the deep learning model and to assess generalizability to other areas, such as diagnosis, etiology, and prognosis.

## Introduction

### Background and Significance

With roughly 95 clinical trials published per day, the biomedical literature is increasing at a very rapid pace, imposing a significant challenge to the practice of evidence-based medicine. However, only 1% of studies in the biomedical literature meet minimum criteria for scientific quality [1] and most published research findings are eventually shown to be false [2]. As a result, a major barrier to the practice of evidence-based medicine is efficiently finding the relatively small number of scientifically sound studies on a given clinical topic. Systematic reviews and meta-analyses attempt to summarize the available evidence on a given clinical question aiming for near perfect recall. However, systematic reviews are often not available and become quickly outdated. Therefore, clinicians may benefit from access to the latest evidence from high-quality clinical trials before they are included in systematic reviews.

For over two decades, the *Clinical Query* filters have been the state-of-the-art approach to retrieve scientifically sound clinical studies from the primary literature, both for the development of systematic reviews and point-of-care decision support [3,4]. The Clinical Query filters consist of Boolean search strategies based on textwords and Medical Subject Headings (MeSH) terms that have been developed and validated through a systematic approach [5]. The search textwords and MeSH terms used in the Clinical Query filters reflect widely accepted criteria for scientifically sound clinical studies, such as “clinical trial,” “random allocation,” and “randomized controlled trial [Publication Type].” Although initially developed in the 1990s, the Clinical Query filters have been updated over time and the recall and precision of the filters developed in 2000 did not significantly change a decade later [6]. Clinical Query filters for several topics are available in PubMed and several other bibliographic biomedical databases, with focuses on areas such as therapy, diagnosis, etiology, and prognosis, and these are tuned for precision or recall. A limitation of the Clinical Query filters is their dependency on MeSH terms, which are added to PubMed citations 23 to 177 days after an article is published (according to a previous study [7]) and 17 to 328 days according to our more recent analysis. In addition, there is room for improvement, especially in terms of retrieval precision.

Previous studies investigated the use of machine learning approaches to automate the retrieval of scientifically sound studies [8-10]. Features used in those studies included bibliometrics (eg, citation count, impact factor), words in the article title and abstract, MeSH terms, Unified Medical Language System (UMLS) concepts, and semantic predications. Although the results of machine learning studies were promising, they had important limitations that precluded wide adoption in practice, such as a requirement for significant feature engineering (eg, UMLS concepts, bibliometrics), reliance on proprietary and time-dependent features (eg, MeSH index, citation counts), and potential overfitting to a particular dataset.

In the present study, we investigated a deep learning approach for the retrieval of scientifically sound treatment studies from PubMed. To overcome limitations of previous methods, we focused on an approach that requires very little feature engineering and does not rely on proprietary or time-dependent features. We then compared the performance of a deep learning model with state-of-the-art PubMed search strategies against Clinical Hedges, a rigorous gold standard of over 50,000 studies that were systematically rated for scientific quality according to rigorous criteria [5].

### Deep Machine Learning

Recent advances in machine learning have led to dramatic improvements in the abilities of computers to mimic human activities. Many of these improvements leverage “deep learning,” and embody neural-networks with many nodes that are fully connected across layers of the network. In the context of supervised deep learning, which we utilized here, such a network is trained by providing many examples of the objective to classify, as well as many counter examples.

### Deep Neural Networks

A Deep Neural Network (DNN) is a fully connected set of “layers,” each of which contains a node that encodes information in the form of a weight associated with a particular feature of the input data. By “connected” we mean that the nodes of each layer connect with the nodes of the next. A DNN is considered “deep” because it can contain many such connected nodes and/or layers, thereby encoding a significant amount of information in the weights applied to the input of each layer.

In the case of text categorization, the input to the network is a set of words (or “word embeddings” described below). Each successive layer of the DNN applies some transformation to the words in the form of linear algebraic operations that progressively encodes more granular features of the data [11,12]. A supervised DNN, such as our approach, requires that each input (eg, set of words) is associated with a class such that the DNN will learn how to associate the words with each class in order to predict the class for newly unseen sets of words. As with most machine learning approaches, the input text can be transformed in a number of ways. In the case of text classification, such transformations could include adding extraneous information such as bibliographic and author information. This process of designing and applying features to optimize classification is known as *feature engineering*.

Although potentially useful, feature engineering is challenging: it may require significant manual effort and introduces the risk that certain features will be too specific to the training data or may even be unavailable. As we discuss below, leveraging the MeSH terms used to index articles in PubMed can certainly help in a task such as ours, but there is no guarantee that such information is available for an article in a timely manner.

Therefore, we opted for an “end-to-end” machine solution. In end-to-end approaches, the DNN is trained solely on the inputs and classes with minimal or no feature engineering. Minimal features are those that are task- and domain-agnostic, such as converting words to lower case, removing stopwords, and stemming. Potential advantages of such an approach include: (1) simpler design, therefore strong results are more likely indicative that the DNN is detecting textual signal, rather than an arcane feature; (2) no reliance on external factors, such as features that may not be timely available; and (3) mitigation of concept drift, since the training features may misalign from those available when a model is deployed. Therefore, end-to-end systems provide a strong justification for a first approach in classification tasks.

### Recurrent Neural Networks and Convolutional Neural Networks

In this study, we utilized a particular deep-learning neural-network known as Convolutional Neural Network (CNN), following the approach of Kim [13]. To some extent, Kim’s CNN architecture has become a *de facto* standard for text classification. CNNs analyze text using sliding word windows of specified sizes. Each sliding word window generates a set of real-valued vectors. Generally, each word or even character is associated with a “word-embedding,” which is a low-dimension real-valued vector that represents the semantic space for the word [14]. Therefore, as each term is associated with a vector, each sliding word window then represents a matrix. Each sliding word window is then passed through an activation function, and a “max pooling” is applied such that only the maximum value is kept from the set of values produced by the activation function, as applied to the window. That is, each window is associated with its single, maximal value outputted by the activation function. These maximal values are concatenated together to form their own vector representing the set of windows. This set of concatenated values forms the next layer, which is then passed to the final layer, which includes the decision-making activation function (such as Softmax, as described below).

**Figure 1:**
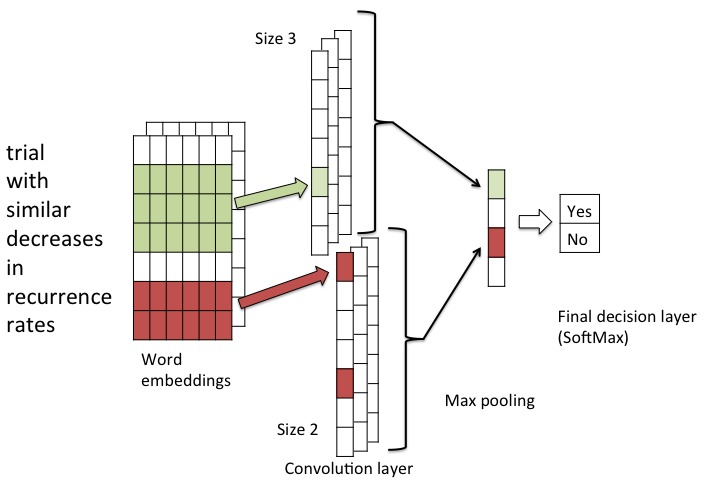
Example of a Convolutional Neural Network.

An example of a CNN is shown in Figure 1. From left to right, we see one set of input words and their word embeddings, which forms the initial input matrix. This network uses two sets of sliding windows, one of size two and one of size three. These sets of sliding windows produce the convolutional layer, transforming the sliding window’s features into new feature values, which are then pooled such that only the maximum value is kept (the “max pooling”). Finally, the max-pooled values are passed through the fully connected final (output) layer, which uses Softmax to assign a probability of class membership (shown as “yes” or “no” for binary class membership). While this approach may appear “shallow,” it has been shown to be effective, becoming one of the most popular architectural choices for CNN [13].

Another popular approach for text-analysis tasks are Recurrent Neural Networks (RNNs). In contrast to CNN’s sliding-windows, which treat phrases somewhat independently, RNNs are well suited for language tasks where the classification of a particular piece of text depends on the surrounding text. For instance, RNNs are well suited for part-of-speech tagging or machine translation, which have a strong dependency on the particular word order. However, because they must consider order dependencies, they are not as appropriate for tasks such as ours. In fact, in a head-to-head comparison between CNNs and RNNs for natural language processing tasks, Yin et al [15] found that CNNs are particularly well suited for so called “keyphrase recognition” tasks such as text classifications or sentiment analyses. Furthermore, CNNs were found to be up to five times faster than RNNs [15], which is important in real-world tasks such as ours where the goal is to classify an extremely large corpus, such as PubMed, in a reasonable amount of time.

### Deep Neural Network Optimization (How It Learns)

The main learning for a neural network involves “forward propagation” and “backward propagation.” In forward propagation, inputs are translated into features by transforming the inputs into real-valued vectors of fixed sizes. These vectors (eg, “layers”) are combined with weights and passed through an activation function that summarizes the contribution of each feature of the vector and its weight. Layers are connected to one another such that the values from the activation function of the current layer become the inputs to the next layer. Therefore, the “forward propagation” starts with input and passes activation values from layer to layer until the final layer, which outputs some decision vector. In our case, this final output function is a sigmoid activation function, which can assign probability to class membership. In “backward propagation” the final classification decision is compared with the known result from the training data and errors are propagated backward through the network, from the output layer to the input layer. Each weight is updated according to its contribution to the decision accuracy via gradient descent.

In the context of CNN, one can interpret the various passes through “forward” propagation as applying weights to different “chunks” of the text input, and “backwards” propagation as adjusting those weights to make the fewest errors in predicting the class of the input text. Within the context of DNN, since optimization is essentially a weight adjustment process, the higher the number of nodes and layers, the more weights must be adjusted to find the optimal classifier, which requires more training data. Conversely, more weights and layers may improve classification. Therefore, part of DNN design is to identify optimal parameter choices and how to deal with overfitting. In our case, we used a technique called dropout regularization, which randomly prevents nodes from participating in a classification decision for a given training input, so the model does not overfit by learning to simply rely on a particular node.

Other optimizations include which mathematical operations to choose for the propagation; this is called the “activation function” (ie, how a node produces a score given the weight and input). Different choices can result in different DNN behavior; some activation functions are more robust than others, while some can make the training process exceedingly long. We chose the Rectified Linear Unit (ReLU) for our activation function, as it provides an efficient mechanism to build robust and accurate CNNs. The choice of ReLU is quite common in tasks such as ours. Finally, within the context of CNN, it is common to provide a down-sampling between layers, which helps control overfitting and makes training more efficient. The most common approach is max pooling, which we use in our approach.

Therefore, the training of a network involves multiple passes of forward propagation followed by backward propagation. It is common to call each iteration over all the training data an “epoch.” The model generally stops this training process at a fixed number of epochs or when the metric of success appears to have reached some maximal value.

### Clinical Hedges

Clinical Hedges is a database previously developed by the Hedges Group at McMaster University, used to develop and evaluate the Clinical Query filters [5] and previous machine learning approaches [8] that retrieve scientifically sound clinical studies from PubMed. The database has 50,594 articles published in 170 clinical journals. All articles were manually annotated by highly-calibrated information science experts according to type (eg, etiology, prognosis, diagnosis, prevention, therapy, clinical prediction) and whether or not each study met prespecified and experimentally validated methodological criteria for scientifically sound clinical research. The criteria and process used to rate the articles in Clinical Hedges are described elsewhere [5]. In summary, criteria for scientifically sound studies on treatment interventions include random allocation of study participants, clinically relevant outcomes, and at least 80% follow-up of study participants.

## Methods

### Overview of the Approach

Overall, our approach consisted of (1) training and testing deep learning models with a large and noisy dataset obtained automatically through PubMed searches based on the Clinical Query treatment filter, and (2) evaluating the performance of the resulting model against Clinical Hedges as a gold standard.

Specifically, the study method consisted of the following steps, which are described in more detail in the sections below: (1) preparation of a dataset for training the deep learning models, (2) training and tuning deep learning models, (3) comparison of the deep learning approach with state-of-the-art search filters and McMaster’s textword filter in terms of precision and recall, and (4) analysis of deep learning performance in terms of precision at several levels of K retrieved citations.

### Preparation of Training Dataset

**Figure 2:**
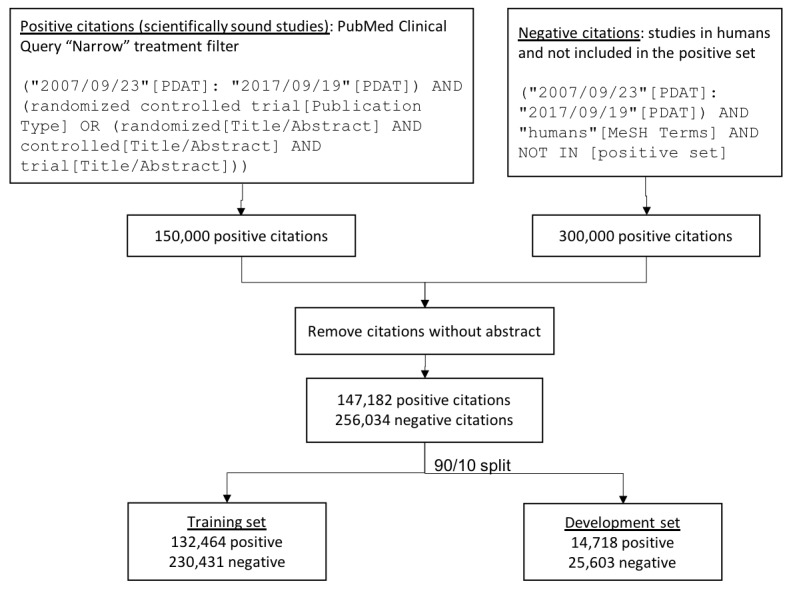
Datasets used for training and testing the deep learning models. The PubMed Clinical Query “Narrow” treatment filter was used as a surrogate to identify positive (scientifically sound) studies. The resulting dataset was split into training and development sets using a 90/10 ratio.

The training/testing dataset consisted of 403,216 positive and negative citations retrieved from PubMed. To retrieve *positive studies* (ie, scientifically sound), we used the Clinical Queries treatment filter tuned for precision (“narrow” filter; Figure 2). In previous studies, this filter yielded 93% recall and 54% precision for scientifically sound treatment studies in the Clinical Hedges gold standard [5]. Therefore, this search strategy was used as a surrogate for retrieving a large dataset of scientifically sound studies that are similar to the ones in the Clinical Hedges gold standard. Although this approach produced a rather noisy training set (close to half of the positive samples were false-positives), the CNN approach is resilient to handle noisy data as long as there is sufficient training data. To retrieve *negative studies* (ie, not scientifically sound), we retrieved studies conducted in humans which were not retrieved by the “positive” search strategy above.

The strategies were limited to retrieve a maximum of 150,000 and 300,000 citations respectively to yield a dataset with one third positive and two thirds negative citations. Both strategies were limited to citations published between 2007 and 2017. Citations without an abstract were removed. The search strategies were executed with PubMed’s eUtils application program interface. The resulting dataset contained 147,182 positive and 256,034 negative citations (Figure 2).

### Training and Tuning Deep Learning Models

Deep learning models were trained using 90% of the citations in the dataset, with the remaining 10% used as a “development” set (Figure 2). As the training/development split was randomly generated, the development set maintained a similar proportion of positive to negative instances as the training set. To build model inputs, we concatenated the title with the abstract, removed stopwords, and kept the first 650 tokens of the remaining words.

As mentioned in the *Deep Machine Learning* section, our model follows the well-accepted approach of applying CNNs for text classification. The first layer applies character embedding to the words, so that words outside of the known vocabulary can be included for prediction. The character embeddings are then combined with word embeddings (built from the training data), to capture semantic similarity. This input is passed into our model, which contains two convolutional layers: one for sliding word windows of size two and one for word windows of size three. Each convolutional layer contains 512 filters associated with it. We apply a ReLU unit to the convolutional layers and pass them through a max pooling procedure. The resulting max-pooled features are then concatenated into a single layer. The max-pooled layer is passed to the next layer which consists of 512 units (fully connected), to which we apply a Softmax activation function to predict the probability of a citation belonging to either class. We then take the Argmax of the Softmax predictions as the predicted class. We ran this model with dropout regularization of 0.5 (to prevent overfitting) for 30 epochs. Hyper-parameters were chosen experimentally based on maximized precision on the training data.

### Comparison of the Deep Learning Approach With State-of-the-Art PubMed Search Strategies

**Figure 3:**
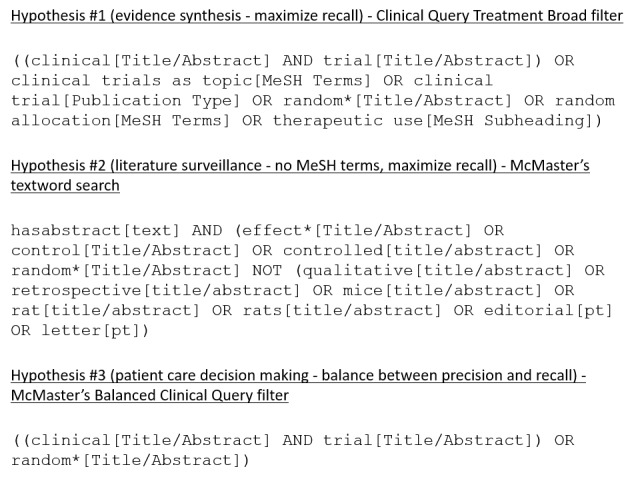
Search strategies used to retrieve scientifically sound treatment studies in comparison with the deep learning model.

We tested three hypotheses that reflect the requirements imposed by different information retrieval scenarios. The first scenario consisted of search strategies to support the development of evidence-based syntheses, such as systematic reviews and clinical guidelines [16]. In this scenario, there is a requirement for near perfect recall. The hypothesis for this scenario was that the deep learning approach would yield equivalent recall with higher precision for scientifically sound treatment studies compared with the PubMed Clinical Queries Broad filter, which has almost perfect recall (Figure 3).

The second scenario reflects the need to retrieve recent studies, such as in literature surveillance efforts to identify new evidence to update existing systematic reviews and clinical guidelines [17-19]. Since Clinical Query filters depend partially on MeSH terms and publication type, they are less effective for literature surveillance. Instead, search strategies based on terms in the citation title and abstract are preferred. The hypothesis for this scenario was that the deep learning approach would yield equivalent recall but higher precision for scientifically sound treatment studies compared with a textword search strategy provided by the Clinical Hedges group from McMaster University (Figure 3).

The third scenario represents clinicians searching the literature for evidence to meet clinicians’ information needs that are raised in the care of a specific patient [20]. In this scenario, trading a small loss in recall for substantial gains in precision is acceptable. We hypothesized that the deep learning approach would yield equivalent recall but higher precision for scientifically sound treatment studies compared with McMaster’s Balanced Clinical Query filter, which uses a combination of textwords, MeSH terms, and publication types (Figure 3).

**Figure 4:**
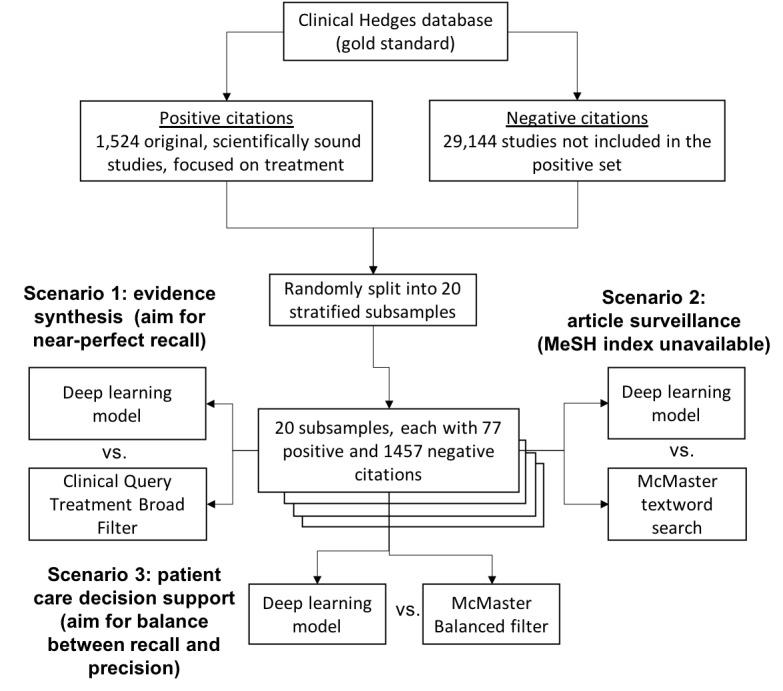
Evaluation method, including comparisons between the deep learning approach and Boolean searches focused on three different information retrieval scenarios.

The Clinical Hedges gold standard was used to test the three hypotheses. For positive citations, we retrieved 1524 original scientifically sound studies, with a focus on treatment, from the Clinical Hedges database. For negative citations, we retrieved 29,144 treatment studies from Clinical Hedges that were not in the positive set. For statistical analyses, we split the resulting dataset into 20 random subsamples, which were stratified to ensure a balanced ratio of positive and negative citations in each subsample. Measures of precision, recall, and F-measure were obtained for the four approaches on each of the 20 subsamples (Figure 4). Last, we ranked the output of the deep learning model according to its probability score and obtained measures of precision at several levels of top K citations (10, 20, 50, 100, 200, 300, and 500).

### Statistical Analysis

Classification performance was measured according to the average precision and recall across 20 data samples. We used the paired Student t-test to test the significance of the differences in recall and precision between the two approaches in each experiment, with the significance level set at 0.05.

## Results

The results are organized according to the three information retrieval scenarios and study hypotheses.

### Scenario 1 - Development of Evidence-Based Syntheses

Table 1 shows the results of the comparisons for Scenario 1, which requires near perfect recall. We tested the hypothesis that *the deep learning approach yields equivalent recall with higher precision for scientifically sound treatment studies compared with the PubMed Clinical Queries Broad filter*. The Clinical Queries Broad filter had statistically significantly higher recall than the deep learning model (98.4% vs 96.9%; *P*=.002), although the difference was small (-1.6%) and likely marginal in practice, depending on the use case. The deep learning model had significantly higher precision than the Clinical Queries Broad filter, with a +12.2% absolute difference (34.6% vs 22.4%; *P*<.001).

### Scenario 2 - Literature Surveillance

Table 2 shows the results of the comparisons for Scenario 2, which requires retrieval of recent studies prior to MeSH indexing. We tested the hypothesis that *the deep learning approach yields equivalent recall but higher precision for scientifically sound treatment studies compared with a textword search strategy*. The deep learning model was equivalent to McMaster’s textword search in terms of recall (97.1% vs 96.9%; *P*=.57); and had significantly higher precision than the textword search (34.6% vs 28.5%; *P*<.001).

### Scenario 3 - Patient Care Decision Support

Table 3 shows the results of the comparisons for Scenario 3, in which trading a small loss in recall for gains in precision is acceptable. We tested the hypothesis that *the deep learning approach yields equivalent recall but higher precision for scientifically sound treatment studies compared with McMaster’s Balanced Clinical Query filter.* Compared with the McMaster Balanced treatment filter, the deep learning model had similar recall (96.9% vs 97.0%; *P*=.63), but lower precision (34.6% vs 40.9%; *P*<.001; Table 3).

### Precision at K

**Figure 5:**
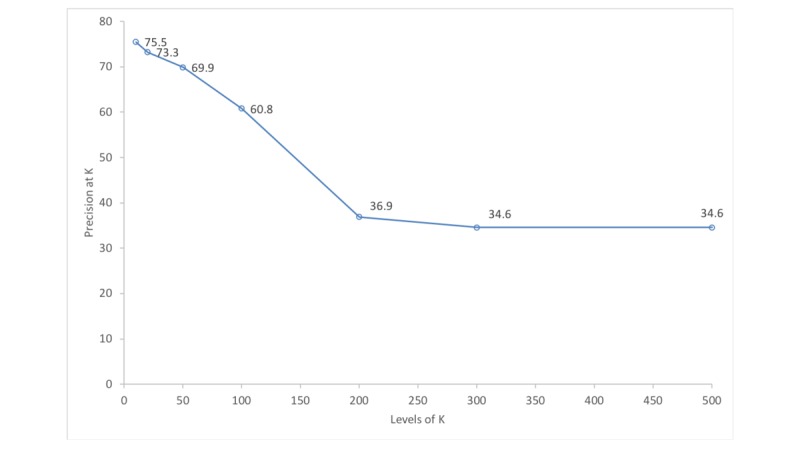
Average precision of the deep learning model at different levels of top K citations.

The precision at K curve for the ranked output of the deep learning model showed that precision ranged from 75.5% to 61% among the top 10 to top 100 citations and only decreased substantially after the top 200, 300, and 500 citations (Figure 5).

**Table 1:** Average recall, precision, and F-measure of the deep learning model and Clinical Query Broad filter according to the Clinical Hedges gold standard (N=20).

Parameter	Deep learning (%)	CQ^a^ broad (%)	*P* value
Recall	96.9	98.4	<.001
Precision	34.6	22.4	<.001
F-measure	51.0	36.5	<.001

^a^CQ: PubMed Clinical Query Treatment filter

**Table 2:** Average recall, precision, and F-measure of the deep learning model and McMaster’s textword search according to the Clinical Hedges gold standard (N=20).

Parameter	Deep learning (%)	Textword search (%)	*P* value
Recall	96.9	97.1	.57
Precision	34.6	11.8	<.001
F-measure	51.0	21.0	<.001

**Table 3:** Average recall, precision, and F-measure of the deep learning approach and McMaster’s Balanced Treatment filter according to the Clinical Hedges gold standard (N=20).

Measure	Deep learning (%)	McMaster’s CQ^a^ balanced filter (%)	*P* value
Recall	96.9	97.0	.63
Precision	34.6	40.9	<.001
F-measure	51.0	57.5	<.001

^a^CQ: PubMed Clinical Query Treatment filter

## Discussion

### Significant Findings

To our knowledge, this is the first study to investigate the use of deep learning techniques to identify reports of scientifically sound studies in the biomedical literature in three different information-seeking scenarios. The deep learning approach performed reasonably well compared with state-of-the-art search filters, especially for literature surveillance. For evidence synthesis, the deep learning approach had slightly lower recall (-1.6%), but significantly higher precision (+12.2%) than the PubMed Clinical Query Broad treatment filter. For literature surveillance, the deep learning approach had equivalent recall to McMaster’s textword filter, but significantly higher precision (+22.2%). For patient care decision-making, the deep learning model had similar recall, but lower precision (-6.3%) than McMaster’s Balanced filter. Strengths of the study methodology include the use of a very large training set, comparison with state-of-the-art search strategies, and evaluation with a rigorous gold standard which was completely independent from the training set.

The proposed deep learning approach has three main potential benefits compared with previous approaches. First, unlike previous machine learning approaches, which depend on features that are not always openly and contemporaneously available (eg, MeSH terms, citation counts, journal impact factors), the proposed deep learning approach only uses citation title and abstract, which are available as soon as citations are entered in PubMed. Although full-text articles could be added as features in an attempt to improve performance, obtaining access to the full-text of all articles indexed in PubMed is impracticable since most journals do not provide open access to full-text. To assess the potential duration of delays for literature surveillance strategies based on MeSH filters, we determined the time between the date of creation of the article record in PubMed (CRDT) and the date of posting of MeSH terms (MHDA) for 107 journals (55,237 articles) in the McMaster PLUS database, from which the Clinical Hedges database was derived. The mean delay in MeSH indexing per journal was 162 days (95% CI 157-167), with a range of 17 to 328 days. Indexing intervals for journals were inversely correlated with journal impact factors (for 2016), but the correlation was relatively weak (-0.38; CI -0.199 to -0.517). As a second benefit, the deep learning model provides a ranked output with 70% or higher precision among the top 50 citations. This feature could be particularly useful for clinicians in busy clinical settings who are less likely to look beyond the top 20 citations that are displayed in PubMed searches [20,21]. In addition, citation ranking could help with systematic review development, since front-loading “eligible” citations can be used to help train and calibrate citation screeners and prioritize work [22]. Third, the deep learning model obtained reasonable performance despite being trained on a noisy dataset (an estimate of roughly 50% of the positive cases were false-positives). This finding confirms the robustness of the deep learning approach, which is known to be resilient to noisy training data [23].

### Comparison With Prior Work

Previous work applied deep learning to classification tasks in the biomedical informatics domain. Lee [24] classified sentences as belonging to papers that would be included in a systematic review, or those that would not. However, because they did not employ a large-scale training procedure, as we devised here, their results were poor. It is also not clear whether the author focused solely on sentence classification, or document classification, as in our work [24]. Hughes et al [25] applied CNNs to classify sentences according to one of 26 categories, such as “Brain” or “Cancer,” using a similar approach (though a different training procedure) to a different problem. Wang et al [26] used word, dependency, and abstract meaning representation embeddings to extract information on drug-drug interactions from the biomedical literature. Both Nguyen et al [27] and Che et al [28] utilized CNNs to predict risk outcomes, such as hospital readmission, using electronic health record data as an input. As with Hughes et al [25], although applied to different problems, the latter studies demonstrated precedent for using CNN in biomedical text classification.

A polynomial Support Vector Machine classifier based on MeSH terms, publication type, and title/abstract words obtained a recall of 96% and precision of 18% against a gold standard of internal medicine articles included in the American College of Physicians Journal Club [9]. A different study compared Clinical Query filters, machine learning, and algorithms based on citation count and the PageRank algorithm using a gold standard of important literature on common problems in surgical oncology [10]. The PageRank algorithm obtained a precision at the top 10, 20, 50, and 100 citations of 7.8%, 13.0%, 19.9%, and 26.3%, respectively [10]. Overall precision and recall were not reported. More recently, a study by Kilicoglu et al [8] investigated a set of classifiers using features such as MeSH terms, title/abstract words, UMLS concepts, and semantic predications. A Naïve Bayes classifier with these features obtained a recall and precision of 91.4% and 52.5% for treatment studies in the Clinical Hedges database [8]. As discussed above, those previous approaches relied on substantial feature engineering and/or proprietary and time-sensitive features, compromising the use of those approaches in real-time information retrieval systems. In a recent study investigating an approach similar to ours, Marshall et al [29] developed CNN and support vector machine classifiers based on article title and abstract to identify reports of randomized controlled trials (RCTs). The best classifier obtained a recall of 98.5% and precision of 21% [29]. Although the authors also evaluated their classifiers against the Clinical Hedges database, the results cannot be directly compared with our study because their goal was to identify RCTs versus scientifically sound studies (not all RCTs are scientifically sound and not all scientifically sound studies are RCTs). Another difference was that Marshall et al [29] used a training set derived from RCTs identified in Cochrane systematic reviews while we used a dataset obtained using the Clinical Queries Treatment Narrow filter.

### Error Analysis

We analyzed a random sample of 20 false-negatives and 20 false-positives identified by the deep learning model. The majority of the false-negatives (16/20) were likely due to the lack of an explicit description of the study design in the article abstract, which led the deep learning model to miss these articles. Of the 20 false-negatives, the Clinical Query Broad filter was able to correctly identify 14 articles based on MeSH terms and publication type rather than words in the abstract or title. Two approaches can be investigated in future studies to address this problem. First, MeSH terms and publication type could be included as deep learning features. The caveat is that this approach would require feature engineering and would be limited by the time lag of MeSH terms and publication type described above. The second, and perhaps more promising approach, is to include the methods section from the article full-text as an input for deep learning. Since the methods section has many more details on the study methodology than the article abstract, it may lead to more accurate classification of scientifically sound studies.

False-positives were due to two main error categories. First, 7 of 20 cases were marginal articles that partially met quality criteria (eg, RCT without a clinical outcome) and therefore were more difficult to rate (7/20). Second, in 11 of 20 cases the abstract included terms related to high quality methodology but stated these outside the context of the study method (eg, abstract conclusion stating the need for future RCTs, editorial raising the need for RCTs on a specific topic). Approaches to mitigate both types of errors include using the full-text of the methods section as input for the deep learning model and developing separate subclassifiers to detect studies that meet partial quality criteria, and nonoriginal studies (eg, editorials, letters, reviews).

### Limitations

Our study has four important limitations. First, although we focused on deep learning models and optimization strategies that were most likely to produce the best results, we have not exhausted all deep learning optimization possibilities. For instance, new work on RNNs may prove more accurate in document classification tasks [30,31]. We chose to focus our efforts on CNNs because they run more efficiently, given the large scale of our text data, but there is a valid investigation into understanding the trade-offs between speed and accuracy by comparing these methods. We also did not exhaustively search the hyper-parameter space for our CNN. Many of our choices were empirical, as this is the first study, and further efforts might leverage more systematic approaches to hyper-parameter tuning [32]. Second, our approach is meant to be “end-to-end” (ie, text simply enters our pipeline and is classified). This approach is preferable because it does not require significant feature engineering or time-dependent features such as MeSH terms. However, further studies can explore adding richer features into our model to improve performance. For example, since the McMaster’s textword filter has equivalent recall as (but lower precision than) the Clinical Query filters, it is possible that MeSH-based features could improve the precision of our deep learning approach. Third, we have made comparisons with only one textword filter and no other machine learning approaches, since we did not have access to those machine learning classifiers. Comparisons with two of the three previous machine learning approaches are indirect, since those studies did not use Clinical Hedges as a gold standard. Last, we focused on identifying “treatment” studies; further work is needed to verify whether our approach generalizes to other areas, such as diagnosis, etiology, and prognosis.

### Conclusion

We compared deep learning with state-of-the-art search filters to identify reports of scientifically sound studies in the biomedical literature. Overall, the resulting deep learning model compared well with other approaches, especially in scenarios involving recent citations prior to MeSH indexing. Advantages of the deep learning approach include low feature engineering requirements, no dependency on proprietary and time-sensitive features, and the use of a very large training set. Future work is needed to investigate further optimization opportunities and to adapt the deep learning approach to other clinical areas. Deep learning is a promising approach to identifying scientifically sound studies from the biomedical literature and warrants further investigation as a potential alternative for, or supplement to, current search filters.

## Abbreviations

CNN: Convolutional Neural Network
DNN: Deep Neural Network
MeSH: Medical Subject Heading
RCT: randomized controlled trial
ReLU: Rectified Linear Unit
RNN: Recurrent Neural Network
UMLS: Unified Medical Language System
